# Benckiser's hemorrhage: about an uncommon case report

**DOI:** 10.1016/j.ijscr.2022.107128

**Published:** 2022-04-27

**Authors:** Aziz Slaoui, Amine Slaoui, Najia Zeraidi, Amina Lakhdar, Brahim Rhrab, Aicha Kharbach, Aziz Baydada

**Affiliations:** a Gynaecology-Obstetrics and Endoscopy Department, Maternity Souissi, University Hospital Center IBN SINA, University Mohammed V, Rabat, Morocco; b Gynaecology-Obstetrics and Endocrinology Department, Maternity Souissi, University Hospital Center IBN SINA, University Mohammed V, Rabat, Morocco; c Urology B Department, Avicenne Hospital, University Hospital Center IBN SINA, University Mohammed V, Rabat, Morocco

**Keywords:** Benckiser's hemorrhage, Obstetric emergencies, Retroplacental hematoma, MRI, Magnetic Resonance Imaging

## Abstract

**Background:**

Benckiser's hemorrhage is a serious obstetric emergency caused by rupture of one or more umbilical vessels of velamentous insertion, putting the fetus in distress and leading to rapid fetal death through exsanguination in utero. It is an uncommon condition associated with a neonatal mortality rate of 75–100%. This hemorrhage of fetal origin happens most often when the membranes rupture, whether spontaneously or artificially.

This is why prenatal diagnosis via ultrasound can only be beneficial and make it possible to schedule a prophylactic caesarean section before the onset of fetal death as well as other adverse perinatal outcomes.

**Case presentation:**

We hereby present an uncommon case of a 27-year-old female patient, with no antenatal check-ups, who presented to the emergency department for labor-like pain at a gestational age of 32 weeks. On examination, blood pressure was 140/89mmhg. Shortly after her hospitalization, the patient was experiencing steady vaginal bleeding as well as spontaneous rupture of the membranes. Retroplacental hematoma was suspected. It was then quickly decided to do an emergency caesarean section. It was only at the examination of the placenta that the diagnosis was corrected with the visualization of torn velamentous vessels, allowing immediate resuscitation of the newborn and admission in neonatal intensive care unit for blood transfusions.

**Conclusions:**

Detailed prenatal ultrasonography screening for vasa previa in high risk pregnancies prevent the onset of complications related to their rupture. An elective caesarean section should be carried out prior to the onset of labor, most often at 35 weeks of amenorrhea, avoiding rupture of membranes and fetal exsanguination, while taking into consideration the impact of iatrogenic prematurity.

## Highlights

• Routine evaluation of the placental cord insertion site can identify pregnancies with velamentous insertion.
• Prenatal diagnosis makes it possible to schedule a prophylactic cesarean section before the onset of fetal death through exsanguination in utero.
• Diagnosis of Benckiser's hemorrhage allows to orientate towards an immediate neonatal management of hypovolemic shock state.

## 1 Background

Benckiser's hemorrhage is a serious obstetric emergency caused by rupture of one or more umbilical vessels of velamentous insertion. It is a rare condition associated with a neonatal mortality rate of 75–100% [1]. Velamentous cord insertion, which occurs at an incidence of approximately 1% in singleton pregnancies and 15% in monochorionic twin pregnancies, is characterized by an umbilical cord attachment to the membranes surrounding the placenta instead of the central mass [2]. It is strongly associated with vasa praevia, where the umbilical vessels belonging to the fetal circulation cross or run in close proximity to the internal cervical os underneath the presenting part, leaving the vessels vulnerable to rupture, which can lead to fatal fetal exsanguination [2], [3].

This is why prenatal identification via ultrasound can only be beneficial and make it possible to schedule a prophylactic caesarean section, reducing fetal mortality from 33% - 100% in cases of vasa previa not diagnosed antenatally to 3% in cases detected before birth [3], [4].

We hereby present an uncommon case of a patient with no antenatal check-ups, who was admitted to our emergency department at 32 weeks of gestation for risk of preterm labor in a context of gestational hypertension. Shortly after her hospitalization, she presented a sudden vaginal bleeding as well as spontaneous rupture of the membranes. Retroplacental hematoma was suspected. It was then quickly decided to do an emergency caesarean section. It was only at the examination of the placenta that the diagnosis was corrected with the visualization of torn velamentous vessels, allowing immediate resuscitation of the newborn and admission in neonatal intensive care unit for blood transfusions.

## 2 Case presentation

We hereby report the uncommon case of a 27-year-old female patient, gravida 2 para 2, with a first pregnancy complicated by premature delivery at 33 weeks' gestation of a newborn of 1870 g with good psychomotor development, whose second pregnancy is estimated at 32 weeks of gestation. She had no antenatal check-ups. The patient presented to our facility with uterine contractions. Physical examination revealed blood pressure at 140/89 mmHg and a negative urine dipstick for proteinuria. Obstetrical examination revealed fundal height of 26 cm corresponding to 33 weeks, cephalic presenting fetus with regular heart rate with a baseline of 140 beats per minute and a 2 cm dilated cervix with partial effacement. She was therefore admitted to the hospital for risk of premature delivery with incidental findings of gestational hypertension. She was immediately given Tocolytic therapy by nicardipine and an intramuscular injection of 12 mg betamethasone to allow fetal lung maturation. Antibacterial treatment was also carried out with 2 g of ampicillin intravenously. She suddenly presented a sudden vaginal bleeding as well as spontaneous rupture of the membranes during the ultrasound scan.

Given the context of hypertension, the presence of uterine contractions and the sudden vaginal bleeding, the diagnosis of retroplacental hematoma was suspected. It was then quickly decided to do an emergency caesarean section. The operation was performed in less than 15 min and allowed the delivery of a pale male newborn weighing 1340-gram with an Apgar score of 1. Placental examination showed, instead of retroplacental hematoma as we initially suspected, rupture of the vessels running along the membranes connecting the placenta to an aberrant cotyledon. Operative time was 27 min and blood loss were quantified at 200 mL.

The newborn's condition required immediate management by the pediatrician. Cardiopulmonary resuscitation was then initiated and bag mask ventilation was subsequently placed improving oxygen saturation back to 100%. Suction was also used. Thanks to intraoperative diagnosis of Benckiser's hemorrhage, neonatal hypovolemic shock was recognized. Endotracheal intubation with positive pressure ventilation was then initiated, in addition to vascular filling with 20 mL isotonic serum in 1 min via an umbilical venous catheter while waiting for blood transfusion. Due to bradycardia, external cardiac massage for 20 min and cardiac stimulation with adrenaline injections were carried out. At 37 min of life, the newborn recovered a stable hemodynamic state and benefited from a transfusion of 20 mL per kg of group O-negative red blood cells. After 7 days in the neonatal intensive care unit and 3 weeks in the neonatal unit, the newborn was discharged from the hospital.

## 3 Discussion

The first case of velamentous cord insertion was observed in 1766 and was first described in 1773 by Wrisberg et al., in the comments of the Goettingen Society [5]. It was not until 1801 that Lobstein et al. first made the connection between the velamentous insertion of the umbilical cord and the risk of fetal hemorrhage leading to rapid death, due to damage to these vessels during rupture of the membranes [6]. However, it was not until 1831 that the German Robertus Benckiser in his inaugural dissertation at the University of Heidelberg presented the first clinical case of the death of a newborn by hypovolemic shock secondary to rupture of pre-vaginal vessels [7]. Following this presentation, his name was attached to this syndrome in the European and later international literature. Since then, publications have become more frequent and more precise, but are still rare due to the low frequency of this feature. A few hundred cases have been published in the literature over two centuries.

There is no such thing as a Benckiser hemorrhage without velamentous insertion vessels; and the frequency of these hemorrhagic events, although difficult to assess, is estimated to be about one case per 50 velamentous insertions [8]. The pathophysiology of velamentous cord insertion is poorly understood and several theories have been developed [9], [10]. The polarization theory is based on an abnormal implantation of the blastocyst stage embryo which is not facing the endometrium but oblique or opposite to it. This would force allantoic vessels to develop on the membranes to reach the future placentation site. Whereas the trophotropism theory, that was described by Strassmann et al. [11] in 1902 and reported by Duchatel et al. [10], would explain both the apparent displacement of the placenta during pregnancy and the more frequent velamentous insertion of the umbilical cord in case of a low-lying placenta. According to this theory, the placenta develops like the roots of a tree in the direction where the nutritional conditions are most satisfactory.

Prenatal diagnosis of vasa previa can be made by ultrasound, MRI, amnioscopy, palpation of the vessels by digital vaginal examination, as well as by finding fetal blood in the intrapartum vaginal blood. Gianopoulos et al. [12] were the first to report in 1987 a case of ultrasound diagnosis of a previa vessel between the placenta and an aberrant cotyledon near the internal cervical os. Nomiyama et al. [13] performed a study in 1998 of 587 fetuses in the second trimester of pregnancy and the cord insertion was visualized in 99.8% of cases. This search required about 1 min for each case with systematic endovaginale ultrasound and with a sensitivity close to 100%, a specificity of 99.8%, a positive predictive value of 83% and a negative predictive value of 100%. These rates were confirmed in 2006 by Ameryckx et al. [14]. The differential diagnosis, mainly funic presentation (loops of umbilical cord lying over the cervix), is made thanks to the persistence of the images with the mobilization of the fetus and while having the patient change position in case of vasa previa. They will also remain in the same location on repeated examinations [15]. Postnatal diagnosis can be made at delivery by macroscopic examination showing ruptured vessels running freely through the membranes and absence of insertion of the umbilical cord on the placenta, but on the membranes, more than two centimeters from the placental edge.

As the occurrence of spontaneous rupture of membranes cannot be predicted, the Society of Obstetricians and Gynaecologists of Canada recommends that, if diagnosed early, antenatal corticosteroid therapy should be given at 28–32 weeks of amenorrhea, and that patients should be admitted to a level 3 maternity from 30 to 32 weeks of amenorrhea, but does not give an ideal term for a scheduled caesarean section [16]. Indeed, the term at which it should be performed is still debated and there are no studies with a sufficient level of evidence to answer this question. In a first study [17], 61 cases of vasa previa diagnosed antenatally were compared to 94 cases diagnosed at birth. Survival rate was better in antenatal diagnosed cases (97% versus 44%; *p* < 0.001) with an earlier term birth (34.6 SA versus 38.2 SA; p < 0.001). This descriptive study does not allow us to determine the ideal term of birth, but the authors recommend 35 weeks of amenorrhea, or even earlier in the case of rupture of the membranes, significant bleeding or labor. Another series of 60 cases of vasa previa [18] shows that after 39 weeks of amenorrhea the risk of emergency caesarean section is high with a poor neonatal prognosis. More recently, the Royal College of Obstetricians and Gynaecologists has published guidelines [19] in which it recommends a planned caesarean section for antenatal diagnosed vasa previa at 34–36 weeks of amenorrhea in asymptomatic women.

Our case is interesting for two main reasons. The first being the initial mistaken diagnosis of retroplacental hematoma given on the basis of uterine contractions associated with Gestational hypertension of fortuitous discovery, which could subsequently be attributable to preterm delivery symptoms. It was only the macroscopic examination of the placenta in the operating room that led to correct the diagnosis to Benckiser's hemorrhage. The second interest of our case lies in the importance of a good interdisciplinary communication between the obstetrician and the pediatrician which allowed to impute the newborn's distress to the hypovolemic shock allowing rapid management by vascular filling and transfusion, which is not always the case [20].

## 4 Conclusions

Detailed prenatal ultrasonography screening for vasa previa in high risk pregnancies prevent the onset of complications related to their rupture. An elective caesarean section should be carried out prior to the onset of labor, most often at 35 weeks of amenorrhea, avoiding rupture of membranes and fetal exsanguination, while taking into consideration the impact of iatrogenic prematurity.

This work has been reported in line with the SCARE 2020 criteria [21].

## Guarantor of submission

The corresponding author is the guarantor of submission.

## Provenance and peer review

Not commissioned, externally peer-reviewed.

## Funding

There are no funding sources to be declared.

## Availability of data and materials

Supporting material is available if further analysis is needed.

## Consent for publication

Written informed consent was obtained from the patient for publication of this case report and any accompanying images. A copy of the written consent is available for review by the Editor-in-Chief of this journal.

## Ethics approval and consent to participate

Ethics approval has been obtained to proceed with the current study. Written informed consent was obtained from the patient for participation in this publication.

## Registration of research studies

N/A.

## CRediT authorship contribution statement

Aziz SLAOUI: study concept and design, data collection, data analysis and interpretation, writing the paper

Amine SLAOUI: study concept and design, data collection, data analysis and interpretation, writing the paper

Najia ZERAIDI: study design, data collection, data interpretation, writing the paper

Amina LAKHDAR: study design, data collection, data interpretation, writing the paper

Aicha KHARBACH: study design, data collection, data interpretation, writing the paper

Aziz BAYDADA: study concept, data collection, data analysis, writing the paper.

## Declaration of competing interest

The authors declare that they have no competing interests.
